# Phen­yl(pyrrolo[2,1-*a*]isoquinolin-3-yl)methanone

**DOI:** 10.1107/S1600536810017101

**Published:** 2010-05-19

**Authors:** Yun Liu, Hong Jiang

**Affiliations:** a Institute of Chemistry and Chemical Engineering, Xuzhou Normal University, Xuzhou 221116, People’s Republic of China; b Key Laboratory of Biotechnology for Medical Plants of Jiangsu Province, Xuzhou Normal University, Xuzhou, Jiangsu 221116, People’s Republic of China

## Abstract

In the title compound, C_19_H_13_NO, the fused isoquinoline–pyrrole system is planar (r.m.s. deviation =  0.0249] Å) and makes a dihedral angle of 53.73 (9)° with the phenyl ring. An intra­molecular C—H⋯O inter­action generates an *S*(6) ring motif.

## Related literature

For the biological activity of indolizine, see: Olden *et al.* (1991 ▶); Jaffrezou *et al.* (1992 ▶). For our work on the direct one-pot syntheses of pyrrolo[2,1-*a*]isoquinolines, see: Liu *et al.* (2010 ▶). For the preparation of pyrrolo[2,1-*a*]isoquinoline, see: Verna *et al.* (2009 ▶). For bond-length data, see: Allen *et al.* (1987 ▶).
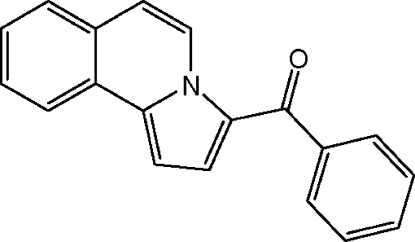


## Experimental

### 

#### Crystal data

C_19_H_13_NO
*M* *_r_* = 271.30
Monoclinic, 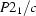

*a* = 28.637 (6) Å
*b* = 4.0400 (8) Å
*c* = 11.824 (2) Å
β = 101.02 (3)°
*V* = 1342.7 (5) Å^3^
*Z* = 4
Mo *K*α radiation
μ = 0.08 mm^−1^
*T* = 295 K
0.30 × 0.20 × 0.10 mm

#### Data collection

Enraf–Nonius CAD-4 diffractometer
Absorption correction: ψ scan (*XCAD4*; Harms & Wocadlo, 1995 ▶) *T*_min_ = 0.976, *T*_max_ = 0.992
2351 measured reflections
2351 independent reflections
1388 reflections with *I* > 2σ(*I*)
3 standard reflections every 200 reflections  intensity decay: none

#### Refinement

*R*[*F* ^2^ > 2σ(*F* ^2^)] = 0.072
*wR*(*F* ^2^) = 0.139
*S* = 1.00
2351 reflections
190 parameters
H-atom parameters constrained
Δρ_max_ = 0.23 e Å^−3^
Δρ_min_ = −0.30 e Å^−3^

### 

Data collection: *CAD-4 Software* (Enraf–Nonius, 1989 ▶); cell refinement: *CAD-4 Software*; data reduction: *XCAD4* (Harms & Wocadlo, 1995 ▶); program(s) used to solve structure: *SHELXTL* (Sheldrick, 2008 ▶); program(s) used to refine structure: *SHELXTL*; molecular graphics: *SHELXTL*; software used to prepare material for publication: *SHELXTL* and *PLATON* (Spek, 2009 ▶).

## Supplementary Material

Crystal structure: contains datablocks I, global. DOI: 10.1107/S1600536810017101/ds2027sup1.cif

Structure factors: contains datablocks I. DOI: 10.1107/S1600536810017101/ds2027Isup2.hkl

Additional supplementary materials:  crystallographic information; 3D view; checkCIF report

## Figures and Tables

**Table 1:** Hydrogen-bond geometry (Å, °)

*D*—H⋯*A*	*D*—H	H⋯*A*	*D*⋯*A*	*D*—H⋯*A*
C19—H19*A*⋯O	0.93	2.31	2.875 (4)	119

**Fig. 1.**
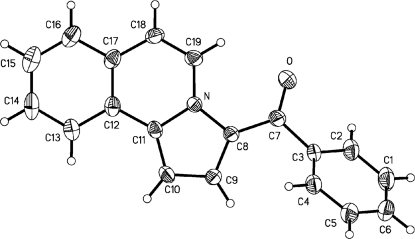
The molecular structure of (I), with atom labels and 50% probability displacement ellipsoids for non-H atoms.

## supplementary crystallographic information

### Comment

The natural and many synthetic indolizines have a diversity of biological activity and are playing an increasingly important role in developing new pharmaceuticals [Olden *et al.*, 1991; Jaffrezou *et al.*, 1992]. Pyrrolo[2,1-a]- isoquinolines are 7,8- benzo- fused indolizines and occur in several marine alkaloids. The synthesis of these structures is drawing much recent research interest [Verna *et al.*, 2009]. In our research work on the direct one pot syntheses of pyrrolo[2,1-a]isoquinolines [Liu *et al.*, 2010], we have prepared the title compound, (I), as one of the products. As part of this study, we have undertaken an X-ray crystallographic analysis of (I) in order to confirm its structure. The bond lengths and angles of the title molecule (Fig. 1) are within normal ranges (Allen *et al.*, 1987). he fused isoquinoline-pyrrole moiety is planar. The dihedral angle between the isoquinoline-pyrrole fused ring and benzene ring is 53.73 (9)°. Although atoms C8, C11 and C19 attached to atom N are all of sp2 hybridization, their different environments cause slight differences in the N—C8, N—C11 and N—C19 bond lengths, and in the C19— N—C11, C19— N—C8 , C11—N—C8 and C10—C11—N angles (Table 1). An intramolecular C—H···O weak hydrogen bond generating an S(6) ring is observed (Table 2). The crystal packing is stabilized by van der Waals forces.

### Experimental

The compound (I) was prepared by the reaction of DMF solution of 2-(2-oxo-2- phenylethyl)isoquinolinium bromide with an excess amount of maleic acid in the presence of TPCD and potassium carbonate. After the reaction was completed, the mixture was isolated by chromatography on a silica gel column after evaporation of the solvent. Single crystals of (I) were obtained by slow evaporation from an petroleum ether-ethyl acetate(3:1) solvent system (yield 80%).

### Refinement

The H atoms were geometrically placed and were treated as riding, with C—H = 0.93Å .

### Crystal data

**Table:**

C_19_H_13_NO	*F*(000) = 568
*M**_r_* = 271.30	*D*_x_ = 1.342 Mg m^−^^3^
Monoclinic, *P*2_1_/*c*	Melting point: 413 K
Hall symbol: -P 2ybc	Mo *K*α radiation, λ = 0.71073 Å
*a* = 28.637 (6) Å	Cell parameters from 25 reflections
*b* = 4.0400 (8) Å	θ = 9–12°
*c* = 11.824 (2) Å	µ = 0.08 mm^−^^1^
β = 101.02 (3)°	*T* = 295 K
*V* = 1342.7 (5) Å^3^	Block, colourless
*Z* = 4	0.30 × 0.20 × 0.10 mm

### Data collection

**Table:**

Enraf–Nonius CAD-4 diffractometer	1388 reflections with *I* > 2σ(*I*)
Radiation source: fine-focus sealed tube	*R*_int_ = 0.0000
graphite	θ_max_ = 25.0°, θ_min_ = 1.5°
ω/2θ scans	*h* = −34→33
Absorption correction: ψ scan (*XCAD4*; Harms & Wocadlo, 1995)	*k* = 0→4
*T*_min_ = 0.976, *T*_max_ = 0.992	*l* = 0→14
2351 measured reflections	3 standard reflections every 200 reflections
2351 independent reflections	intensity decay: none

### Refinement

**Table:**

Refinement on *F*^2^	Secondary atom site location: difference Fourier map
Least-squares matrix: full	Hydrogen site location: inferred from neighbouring sites
*R*[*F*^2^ > 2σ(*F*^2^)] = 0.072	H-atom parameters constrained
*wR*(*F*^2^) = 0.139	*w* = 1/[σ^2^(*F*_o_^2^) + (0.015*P*)^2^ + 2.250*P*] where *P* = (*F*_o_^2^ + 2*F*_c_^2^)/3
*S* = 1.00	(Δ/σ)_max_ < 0.001
2351 reflections	Δρ_max_ = 0.23 e Å^−^^3^
190 parameters	Δρ_min_ = −0.29 e Å^−^^3^
0 restraints	Absolute structure: (*XCAD4*; Harms & Wocadlo, 1995)
Primary atom site location: structure-invariant direct methods	

### Special details

**Table:**

Geometry. All esds (except the esd in the dihedral angle between two l.s. planes) are estimated using the full covariance matrix. The cell esds are taken into account individually in the estimation of esds in distances, angles and torsion angles; correlations between esds in cell parameters are only used when they are defined by crystal symmetry. An approximate (isotropic) treatment of cell esds is used for estimating esds involving l.s. planes.
Refinement. Refinement of *F*^2^ against ALL reflections. The weighted *R*-factor wR and goodness of fit *S* are based on *F*^2^, conventional *R*-factors *R* are based on *F*, with *F* set to zero for negative *F*^2^. The threshold expression of *F*^2^ > σ(*F*^2^) is used only for calculating *R*-factors(gt) etc. and is not relevant to the choice of reflections for refinement. *R*-factors based on *F*^2^ are statistically about twice as large as those based on *F*, and *R*- factors based on ALL data will be even larger.

### Fractional atomic coordinates and isotropic or equivalent isotropic displacement parameters (Å^2^)

**Table:**

	*x*	*y*	*z*	*U*_iso_*/*U*_eq_	
N	0.22455 (9)	0.5534 (7)	0.9319 (2)	0.0382 (7)	
C11	0.19331 (11)	0.4146 (9)	0.9955 (3)	0.0402 (9)	
C7	0.31218 (12)	0.5686 (10)	0.9370 (3)	0.0485 (10)	
O	0.30873 (9)	0.6625 (9)	0.8377 (2)	0.0725 (10)	
C12	0.14286 (12)	0.4449 (10)	0.9532 (3)	0.0447 (9)	
C3	0.36071 (11)	0.5108 (10)	1.0092 (3)	0.0453 (9)	
C8	0.27112 (11)	0.4828 (9)	0.9873 (3)	0.0402 (9)	
C19	0.20843 (12)	0.7328 (10)	0.8334 (3)	0.0461 (9)	
H19A	0.2302	0.8309	0.7947	0.055*	
C18	0.16185 (12)	0.7680 (10)	0.7927 (3)	0.0513 (10)	
H18A	0.1517	0.8929	0.7265	0.062*	
C2	0.37259 (12)	0.6034 (10)	1.1233 (3)	0.0521 (10)	
H2A	0.3496	0.6935	1.1600	0.062*	
C17	0.12699 (12)	0.6158 (10)	0.8496 (3)	0.0483 (10)	
C9	0.26796 (12)	0.3050 (10)	1.0848 (3)	0.0458 (9)	
H9A	0.2936	0.2284	1.1389	0.055*	
C10	0.22029 (12)	0.2579 (10)	1.0897 (3)	0.0477 (10)	
H10A	0.2086	0.1416	1.1462	0.057*	
C13	0.10950 (12)	0.3043 (10)	1.0117 (3)	0.0528 (10)	
H13A	0.1197	0.1878	1.0798	0.063*	
C14	0.06138 (14)	0.3388 (13)	0.9681 (4)	0.0706 (14)	
H14A	0.0393	0.2498	1.0080	0.085*	
C16	0.07802 (13)	0.6403 (12)	0.8072 (3)	0.0628 (12)	
H16A	0.0672	0.7509	0.7382	0.075*	
C6	0.45267 (14)	0.4265 (12)	1.1284 (4)	0.0711 (13)	
H6A	0.4837	0.3990	1.1684	0.085*	
C15	0.04577 (15)	0.5046 (13)	0.8655 (4)	0.0711 (14)	
H15A	0.0134	0.5238	0.8361	0.085*	
C4	0.39519 (13)	0.3782 (11)	0.9543 (3)	0.0567 (11)	
H4A	0.3875	0.3207	0.8769	0.068*	
C5	0.44071 (14)	0.3323 (12)	1.0152 (4)	0.0684 (13)	
H5A	0.4635	0.2365	0.9792	0.082*	
C1	0.41862 (13)	0.5626 (12)	1.1833 (3)	0.0650 (12)	
H1A	0.4267	0.6263	1.2602	0.078*	

### Atomic displacement parameters (Å^2^)

**Table:**

	*U*^11^	*U*^22^	*U*^33^	*U*^12^	*U*^13^	*U*^23^
N	0.0359 (15)	0.0496 (19)	0.0307 (13)	0.0024 (15)	0.0101 (11)	−0.0003 (15)
C11	0.0396 (19)	0.048 (2)	0.0360 (17)	−0.0023 (18)	0.0148 (15)	−0.0037 (18)
C7	0.046 (2)	0.064 (3)	0.0387 (18)	0.013 (2)	0.0169 (16)	0.007 (2)
O	0.0555 (17)	0.117 (3)	0.0500 (15)	0.0072 (19)	0.0227 (13)	0.0212 (19)
C12	0.0393 (19)	0.050 (2)	0.047 (2)	−0.0018 (19)	0.0148 (16)	−0.013 (2)
C3	0.0328 (18)	0.058 (3)	0.049 (2)	0.0002 (19)	0.0162 (16)	0.007 (2)
C8	0.0398 (19)	0.050 (2)	0.0334 (17)	0.0048 (18)	0.0122 (14)	0.0029 (18)
C19	0.051 (2)	0.057 (3)	0.0318 (17)	0.006 (2)	0.0117 (15)	0.0012 (19)
C18	0.049 (2)	0.064 (3)	0.0399 (19)	0.017 (2)	0.0073 (16)	0.009 (2)
C2	0.043 (2)	0.062 (3)	0.054 (2)	−0.002 (2)	0.0160 (17)	0.000 (2)
C17	0.0379 (19)	0.061 (3)	0.046 (2)	0.008 (2)	0.0071 (15)	−0.010 (2)
C9	0.0394 (19)	0.060 (3)	0.0387 (18)	0.008 (2)	0.0095 (15)	0.0068 (19)
C10	0.045 (2)	0.061 (3)	0.0396 (18)	−0.007 (2)	0.0161 (15)	0.006 (2)
C13	0.046 (2)	0.057 (3)	0.059 (2)	−0.008 (2)	0.0191 (18)	−0.010 (2)
C14	0.041 (2)	0.091 (4)	0.085 (3)	−0.015 (3)	0.026 (2)	−0.028 (3)
C16	0.046 (2)	0.075 (3)	0.064 (2)	0.012 (2)	0.0013 (19)	−0.008 (3)
C6	0.040 (2)	0.082 (4)	0.090 (3)	−0.004 (2)	0.008 (2)	0.022 (3)
C15	0.042 (2)	0.083 (4)	0.086 (3)	0.003 (3)	0.007 (2)	−0.027 (3)
C4	0.046 (2)	0.064 (3)	0.066 (2)	0.003 (2)	0.0244 (19)	−0.002 (2)
C5	0.048 (2)	0.068 (3)	0.097 (3)	0.006 (2)	0.033 (2)	0.005 (3)
C1	0.048 (2)	0.085 (3)	0.060 (2)	−0.012 (3)	0.0059 (19)	0.003 (3)

### Geometric parameters (Å, °)

**Table:**

N—C19	1.374 (4)	C17—C16	1.399 (5)
N—C11	1.392 (4)	C9—C10	1.390 (4)
N—C8	1.399 (4)	C9—H9A	0.9300
C11—C10	1.382 (5)	C10—H10A	0.9300
C11—C12	1.441 (4)	C13—C14	1.383 (5)
C7—O	1.220 (4)	C13—H13A	0.9300
C7—C8	1.458 (4)	C14—C15	1.383 (6)
C7—C3	1.504 (5)	C14—H14A	0.9300
C12—C13	1.402 (5)	C16—C15	1.368 (6)
C12—C17	1.404 (5)	C16—H16A	0.9300
C3—C2	1.379 (5)	C6—C5	1.371 (5)
C3—C4	1.388 (4)	C6—C1	1.385 (5)
C8—C9	1.376 (4)	C6—H6A	0.9300
C19—C18	1.336 (4)	C15—H15A	0.9300
C19—H19A	0.9300	C4—C5	1.376 (5)
C18—C17	1.444 (5)	C4—H4A	0.9300
C18—H18A	0.9300	C5—H5A	0.9300
C2—C1	1.382 (5)	C1—H1A	0.9300
C2—H2A	0.9300		
			
C19—N—C11	121.6 (3)	C8—C9—C10	109.2 (3)
C19—N—C8	129.8 (3)	C8—C9—H9A	125.4
C11—N—C8	108.5 (3)	C10—C9—H9A	125.4
C10—C11—N	107.6 (3)	C11—C10—C9	107.8 (3)
C10—C11—C12	133.4 (3)	C11—C10—H10A	126.1
N—C11—C12	118.9 (3)	C9—C10—H10A	126.1
O—C7—C8	123.0 (3)	C14—C13—C12	120.0 (4)
O—C7—C3	119.4 (3)	C14—C13—H13A	120.0
C8—C7—C3	117.4 (3)	C12—C13—H13A	120.0
C13—C12—C17	119.5 (3)	C13—C14—C15	120.5 (4)
C13—C12—C11	121.8 (3)	C13—C14—H14A	119.7
C17—C12—C11	118.7 (3)	C15—C14—H14A	119.7
C2—C3—C4	119.8 (3)	C15—C16—C17	121.2 (4)
C2—C3—C7	122.8 (3)	C15—C16—H16A	119.4
C4—C3—C7	117.2 (3)	C17—C16—H16A	119.4
C9—C8—N	106.9 (3)	C5—C6—C1	120.1 (4)
C9—C8—C7	130.8 (3)	C5—C6—H6A	119.9
N—C8—C7	122.1 (3)	C1—C6—H6A	119.9
C18—C19—N	120.8 (3)	C16—C15—C14	120.0 (4)
C18—C19—H19A	119.6	C16—C15—H15A	120.0
N—C19—H19A	119.6	C14—C15—H15A	120.0
C19—C18—C17	121.2 (3)	C5—C4—C3	119.7 (4)
C19—C18—H18A	119.4	C5—C4—H4A	120.2
C17—C18—H18A	119.4	C3—C4—H4A	120.2
C3—C2—C1	120.2 (4)	C6—C5—C4	120.5 (4)
C3—C2—H2A	119.9	C6—C5—H5A	119.7
C1—C2—H2A	119.9	C4—C5—H5A	119.7
C16—C17—C12	118.8 (4)	C6—C1—C2	119.6 (4)
C16—C17—C18	122.5 (4)	C6—C1—H1A	120.2
C12—C17—C18	118.6 (3)	C2—C1—H1A	120.2
			
C19—N—C11—C10	−178.7 (3)	C13—C12—C17—C16	−0.1 (6)
C8—N—C11—C10	−0.2 (4)	C11—C12—C17—C16	178.8 (4)
C19—N—C11—C12	3.5 (5)	C13—C12—C17—C18	178.0 (4)
C8—N—C11—C12	−178.0 (3)	C11—C12—C17—C18	−3.0 (5)
C10—C11—C12—C13	1.4 (7)	C19—C18—C17—C16	−178.2 (4)
N—C11—C12—C13	178.5 (3)	C19—C18—C17—C12	3.8 (6)
C10—C11—C12—C17	−177.5 (4)	N—C8—C9—C10	1.2 (4)
N—C11—C12—C17	−0.4 (5)	C7—C8—C9—C10	−173.2 (4)
O—C7—C3—C2	138.8 (4)	N—C11—C10—C9	0.9 (4)
C8—C7—C3—C2	−46.0 (6)	C12—C11—C10—C9	178.2 (4)
O—C7—C3—C4	−36.8 (6)	C8—C9—C10—C11	−1.3 (5)
C8—C7—C3—C4	138.3 (4)	C17—C12—C13—C14	−1.0 (6)
C19—N—C8—C9	177.8 (3)	C11—C12—C13—C14	−179.9 (4)
C11—N—C8—C9	−0.6 (4)	C12—C13—C14—C15	1.6 (7)
C19—N—C8—C7	−7.2 (6)	C12—C17—C16—C15	0.7 (6)
C11—N—C8—C7	174.4 (3)	C18—C17—C16—C15	−177.3 (4)
O—C7—C8—C9	162.9 (4)	C17—C16—C15—C14	−0.2 (7)
C3—C7—C8—C9	−12.1 (6)	C13—C14—C15—C16	−1.0 (7)
O—C7—C8—N	−10.8 (6)	C2—C3—C4—C5	1.7 (6)
C3—C7—C8—N	174.2 (3)	C7—C3—C4—C5	177.5 (4)
C11—N—C19—C18	−2.9 (5)	C1—C6—C5—C4	1.4 (7)
C8—N—C19—C18	178.9 (4)	C3—C4—C5—C6	−2.2 (7)
N—C19—C18—C17	−0.8 (6)	C5—C6—C1—C2	−0.1 (7)
C4—C3—C2—C1	−0.4 (6)	C3—C2—C1—C6	−0.4 (7)
C7—C3—C2—C1	−175.9 (4)		

### Hydrogen-bond geometry (Å, °)

**Table:**

*D*—H···*A*	*D*—H	H···*A*	*D*···*A*	*D*—H···*A*
C19—H19A···O	0.93	2.31	2.875 (4)	119
